# The Development of Object Recognition Requires Experience with the Surface Features of Objects

**DOI:** 10.3390/ani14020284

**Published:** 2024-01-17

**Authors:** Justin Newell Wood, Samantha Marie Waters Wood

**Affiliations:** 1 Departments of Informatics, Cognitive Science, Neuroscience, Center for Integrated Study of Animal Behavior, Indiana University, Bloomington, IN 47408, USA; 2 Departments of Informatics and Neuroscience, Indiana University, Bloomington, IN 47408, USA; sw113@iu.edu

**Keywords:** controlled rearing, object recognition, newborn, development, line drawing, chick

## Abstract

**Simple Summary:**

Understanding how brains work requires understanding the role of experience in the development of core mental abilities. Here, we show that the development of one core mental ability—object recognition—requires visual experience with the surface features of objects. We found that when newborn chicks were raised with objects containing surface features, the chicks learned to recognize objects across familiar and novel viewpoints. However, when chicks were raised with line drawings of those same objects, the chicks failed to develop object recognition. These findings shed light on the role of experience in early visual development and suggest that certain kinds of experiences are especially important for the development of core mental abilities.

**Abstract:**

What role does visual experience play in the development of object recognition? Prior controlled-rearing studies suggest that newborn animals require slow and smooth visual experiences to develop object recognition. Here, we examined whether the development of object recognition also requires experience with the surface features of objects. We raised newborn chicks in automated controlled-rearing chambers that contained a single virtual object, then tested their ability to recognize that object from familiar and novel viewpoints. When chicks were reared with an object that had surface features, the chicks developed view-invariant object recognition. In contrast, when chicks were reared with a line drawing of an object, the chicks failed to develop object recognition. The chicks reared with line drawings performed at chance level, despite acquiring over 100 h of visual experience with the object. These results indicate that the development of object recognition requires experience with the surface features of objects.

## 1. Introduction

Mature animals have powerful object recognition abilities. For example, after just a brief glimpse of an object, humans can recognize that object across substantial variation in the retinal images produced by the object, due to changes in viewpoint, size, illumination, and so forth (reviewed in [1]). However, the origins of object recognition are still not well understood. What role does early visual experience play in the development of object recognition? Does the development of object recognition require a specific type of visual experience with objects?

Human infants are not well suited for addressing these questions because they cannot be raised in strictly controlled environments from birth. In contrast, controlled-rearing studies of newborn animals can directly probe the role of experience in development. By systematically manipulating the visual experiences provided to newborn animals and measuring the effects of those manipulations on behavioral and neural development, controlled-rearing studies can isolate the specific experiences that drive the development of object recognition. 

Prior controlled-rearing studies with newborn chicks have revealed two types of experiences that are necessary for the development of object perception: slow and smooth experiences with objects [2,3,4,5]. When newborn chicks were reared with virtual objects that changed slowly and smoothly over time (akin to natural objects), the chicks successfully developed object recognition, including the ability to recognize objects across novel viewpoints, backgrounds, and motion speeds. Conversely, when chicks were reared with objects that moved too quickly or non-smoothly, the chicks failed to develop object recognition. Without slow and smooth visual experiences, newborn chicks develop inaccurate object representations. Here, we extend these findings by examining whether the development of object recognition also requires experience with the surface features of objects. The term “surface features” refers to the features (e.g., color, texture, and shading) of the surfaces between the boundaries of an object. 

There are mixed perspectives on the importance of surface features in object recognition. On one hand, a large number of studies have shown that human adults can readily recognize objects depicted in line drawings, which lack surface features such as color, texture, and shading (e.g., [6,7,8,9]). This ability to perceive and understand line drawings emerges early in development. For instance, infants begin showing enhanced attention to lines that depict corners and edges in the first year of life [10], and young children use lines to define the boundaries of objects in their first attempts to depict the world [11]. Humans have also used line drawings to capture scenes since prehistoric times [12,13]. Furthermore, many nonhuman animals can understand line drawings. Chimpanzees can recognize objects presented in line drawings [14,15] and pigeons can recognize line drawings of objects that are rotated in depth, even after exposure to just a single depth orientation [16]. Even insects appear to use line representation to some extent in biomimicry [17]. Together, these studies indicate that the ability to understand line drawings emerges early in development and is shared with a wide range of animals. 

On the other hand, many studies provide evidence that surface features play an important role in object recognition (e.g., [18,19,20,21,22,23]). Human adults can recognize an object faster when the light source remains in the same location compared to when the light source moves [21], and surface features can affect the speed and accuracy of object recognition and object naming [24]. During their first months of life, human infants also rely on the motion of surface features to build object representations (reviewed in [25]). 

In all of the studies cited above, the subjects had acquired months to years of visual experience with real-world objects before they were tested. Thus, these studies do not reveal whether newborn brains can understand line drawings at the onset of vision or whether the development of this ability requires experience with natural visual objects. The present study distinguishes between these possibilities by testing whether newborn chicks can recognize objects presented in line drawings at the onset of vision, in the absence of prior visual experience with natural objects. Specifically, we contrasted the object recognition performance of newborn chicks reared with line drawings of objects versus realistic objects with surface features. The three experiments presented here allow for a direct test of the importance of surface features in the development of object recognition. 

Across three experiments, newborn chicks were reared in strictly controlled environments that contained no objects other than the virtual objects projected on the display walls (input phase). For one group of chicks, the virtual object(s) contained surfaces (Surface Feature Condition), whereas for the other group, the virtual object(s) was a line drawing animation that lacked surfaces (Line Drawing Condition). In the test phase, we then used a two-alternative forced-choice procedure to measure whether the chicks could recognize their imprinted object across familiar and novel viewpoints. If chicks can recognize objects presented in line drawings, then their performance should be high in both conditions. Conversely, if the development of object recognition requires visual experience with the surface features of objects, then the chicks should develop more accurate object recognition abilities in the Surface Feature Condition than the Line Drawing Condition.

In the first experiment, each chick was reared with a single virtual object (either with or without surfaces) that moved through a limited 60° viewpoint range. In the second experiment, we verified the results of Experiment 1 under different rearing and testing conditions by rearing each chick with a single virtual object (either with or without surfaces) that moved through a 360° viewpoint range. Finally, in the third experiment, we tested whether our findings would extend to new 2D object shapes. Across all three experiments, the chicks successfully recognized objects with surface features, but failed to recognize line drawings of those same objects.

### Using Automated Controlled Rearing to Study the Origins of Object Recognition

To examine the role of surface features in the development of object recognition, we used an automated controlled-rearing method [26]. We used controlled-rearing chambers to eliminate any exposure to real-world objects. There are two benefits to using automated methods to probe the origins of visual intelligence. First, automation allows large amounts of precise behavioral data to be collected from each subject. In the present study, each chick’s behavior was recorded continuously (24/7) for up to two weeks, providing precise measurements of their object recognition performance. Second, since computers (rather than researchers) present the stimuli and code the behavior, automation eliminates the possibility of experimenter error and bias [27]. 

We used newborn chicks as an animal model because they are an ideal model system for studying the origins of object recognition [28]. First, newborn chicks can be raised in strictly controlled environments immediately after hatching (e.g., environments containing no real-world objects). As a result, it is possible to control and manipulate all of the chicks’ visual object experiences from the onset of vision. Second, chicks imprint to objects seen in the first few days of life and will attempt to reunite with those objects when separated [29]. This imprinting behavior emerges spontaneously and provides a reliable behavioral assay for measuring chicks’ object recognition abilities. Third, newborn chicks develop high-level object recognition. For example, newborn chicks can solve the visual binding problem, building integrated object representations with bound color–shape features [30]. Chicks can also parse objects from complex backgrounds [31], build view-invariant object representations [26,32], and recognize objects rapidly, within a fraction of a second [33]. 

Finally, studies of chicks can also inform human development because birds and mammals process sensory input using homologous cortical circuits with similar connectivity patterns [34,35,36,37]. The cortical circuits are organized differently in birds and mammals (nuclear vs. layered organization), but the circuits share similar cell morphology, connectivity patterns of input and output neurons, gene expression, and function [34,38,39,40]. Architecturally, avian and mammalian brains share the same large-scale organizational principles. Specifically, their brains are modular, small-world networks. These networks are organized into a connective core of hub nodes that includes visual, auditory, limbic, prefrontal, premotor, and hippocampal structures [41]. The similarities between avian and mammalian brains suggest that controlled-rearing studies of newborn chicks can elucidate both avian and mammalian intelligence.

## 2. Experiment 1

In the input phase Experiment 1, newborn chicks were reared with a single virtual object moving through a limited 60° viewpoint range. In the test phase, we examined whether the chicks could recognize that object across 12 different viewpoint ranges. The chicks were either raised and tested with line drawings or with objects containing surface features. The text describing the methods is partly adapted from [26]. The data from the baseline (surface feature) conditions in Experiments 1, 2, and 3 were published previously in [4,26,42]. In the present study, we directly contrasted chicks reared with line drawings of objects versus objects with surface features.

### 2.1. Materials and Methods

#### 2.1.1. Subjects

Twenty-three domestic chicks of unknown sex were tested. We tested 11 subjects in the Surface Feature Condition [26] and 12 subjects in the Line Drawing Condition. No subjects were excluded from the analyses. The eggs were obtained from a local distributor and incubated in darkness in an OVA-Easy incubator (Brinsea Products Inc., Titusville, FL, USA). After hatching, we moved the chicks from the incubation room with the aid of night vision goggles. Each chick was placed, singularly, in a controlled-rearing chamber.

This research was approved by The University of Southern California Institutional Animal Care and Use Committee.

#### 2.1.2. Controlled-Rearing Chambers

**Figure 1:**
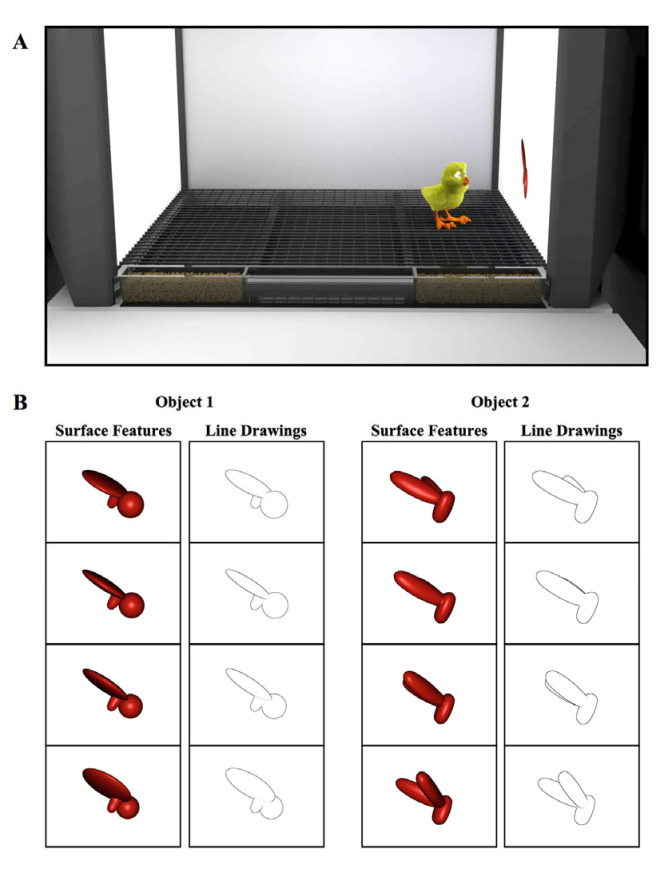
(**A**) Illustration of a controlled-rearing chamber. The chambers contained no real-world objects. To present object stimuli to the chicks, virtual objects were projected on two display walls situated on opposite sides of the chamber. During the input phase (1st week of life), newborn chicks were exposed to a single virtual object either with surface features or without surface features (line drawing). (**B**) Sample images of the virtual objects.

The controlled-rearing chambers (66 cm length × 42 cm width × 69 cm height) were constructed from white, high-density plastic. Each chamber contained no real-world (solid, bounded) objects (Figure 1A). We presented object stimuli to the chicks by projecting animations of virtual objects on two display walls situated on opposite sides of the chamber. The display walls were 19″ liquid crystal display (LCD) monitors with 1440 × 900 pixel resolution. We provided food and water in transparent troughs in the ground (66 cm length × 2.5 cm width × 2.7 cm height). We fed the chicks grain because grain does not behave like an object (i.e., a heap of grain does not maintain a solid, bounded shape). The floors were wire mesh and supported 2.7 cm off the ground by transparent beams.

We embedded micro-cameras in the ceilings of the chambers to record all of the chicks’ behavior (9 samples/s, 24 h/day, 7 days/week). We used automated image-based tracking software (EthoVision XT, version 7, Noldus Information Technology, Leesburg, VA, USA) to track their behavior throughout the experiment. This automated data collection approach allowed us to collect 168 trials from each chick. In total, 7728 h of video footage (14 days × 24 h/day × 23 subjects) were collected for Experiment 1.

#### 2.1.3. Procedure

In the first week of life (input phase), newborn chicks were reared in controlled-rearing chambers that contained a single virtual object. On average, the object measured 8 cm (length) × 7 cm (height) and was displayed on a uniform white background. Eleven of the chicks were imprinted to Object 1 (with Object 2 serving as the unfamiliar object), and twelve of the chicks were imprinted to Object 2 (with Object 1 serving as the unfamiliar object). The objects were modeled after those used in previous studies that tested for invariant object recognition in adult rats [43].

The object moved continuously (24 frames/s), rotating through a 60° viewpoint range about a vertical axis passing through its centroid (Figure 1B). The object only moved along this 60° trajectory; the chicks never observed the object from any other viewpoint in the input phase. The object switched display walls every 2 h (following a 1 min period of darkness), appearing for an equal amount of time on the left and right display wall. In the Surface Feature Condition, the imprinted object had realistic surface features, whereas in the Line Drawing Condition, the imprinted object was a line drawing animation of the object (Figure 1B, see Movie S1 for animations).

In the second week of life (test phase), the chicks received 168 test trials (24 test trials per day). During the test trials, the imprinted object was shown on one screen and an unfamiliar object was shown on the other screen. We expected the chicks to spend a greater proportion of time in proximity to the object that they perceived to be their imprinted object.

For all test trials, the unfamiliar object was presented from the same viewpoint range as the imprinted object shown during the input phase. The unfamiliar object had a similar size, color, motion speed, and motion trajectory as the imprinted object from the input phase. Consequently, for all of the novel viewpoint ranges, the unfamiliar object was more similar to the imprinting stimulus (from a pixel-wise perspective) than the imprinted object was to the imprinting stimulus (for details, see [26]). To recognize their imprinted object, the chicks needed to generalize across large, novel, and complex changes in the object’s appearance on the retina. 

**Figure 2:**
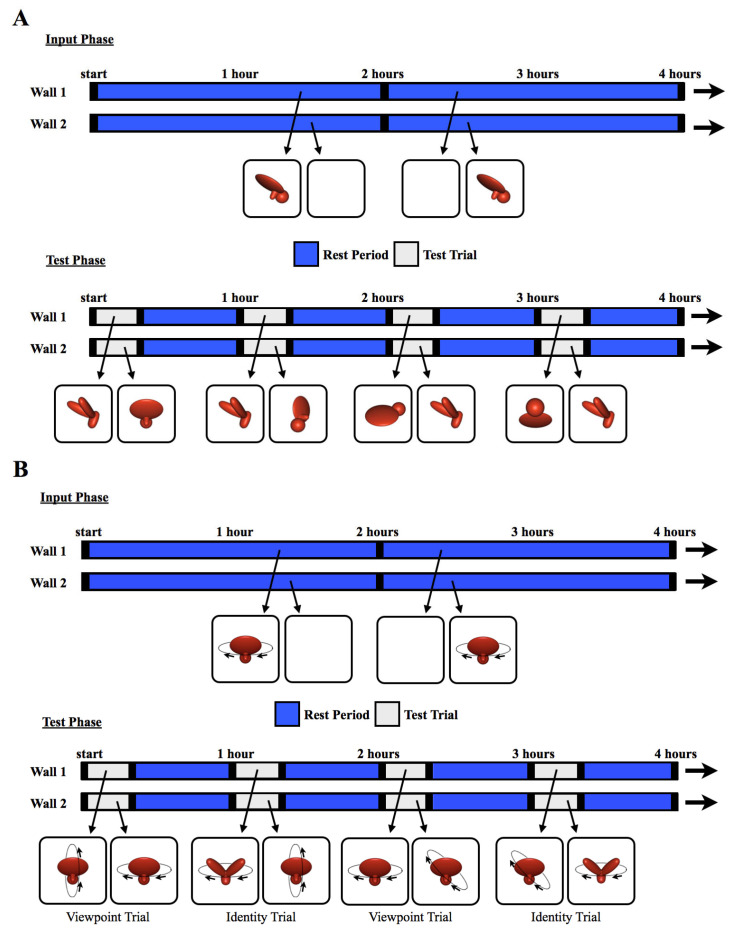
The experimental procedure. The schematics illustrate how the objects were presented for sample 4 h periods during (**A**) Experiment 1 and (**B**) Experiment 2. During the input phase, chicks were exposed to a single virtual object moving through a 60° (Experiment 1) or 360° (Experiment 2) viewpoint range. The object appeared on one wall at a time (indicated by blue segments on the timeline), switching walls every 2 h, after a 1 min period of darkness (black segments). During the test trials, two virtual objects were shown simultaneously, one on each wall, for 20 min per hour (gray segments). The illustrations below the timeline are examples of paired test objects displayed in four of the test trials. Each test trial was followed by a 40 min rest period (blue segments). During the rest periods, the animation from the input phase was shown on one wall, and the other wall was blank. This figure shows the stimuli from the Surface Feature Condition. In the Line Drawing Condition, the chicks were raised and tested with line drawings rather than objects with surface features.

The chicks were tested across 12 viewpoint ranges (11 novel, 1 familiar). Each viewpoint range was tested twice per day. The test trials lasted 20 min and were separated from one another by 40 min rest periods. During the rest periods, the animation from the input phase appeared on one display wall and a white screen appeared on the other display wall. The 12 viewpoint ranges were tested 14 times each within randomized blocks over the course of the test phase. Figure 2A illustrates how the objects were presented across the display walls during the input phase and test phase. In the Surface Feature Condition, the chicks were tested with objects containing surface features, whereas in the Line Drawing Condition, the chicks were tested with line drawings of the objects. In both conditions, the test objects moved continuously through a 60° viewpoint range.

### 2.2. Results

To analyze the chicks’ behavior, the image-based tracking software scored the chick as being in proximity to an object when the chick occupied a 22 × 42 cm zone next to the object. Then, we computed the number of test trials in which chicks preferred their imprinted object over the unfamiliar object. The chick was rated to have preferred their imprinted object on a trial if their object preference score was greater than 50%. The object preference score was calculated with the formula Object Preference Score = Time by Imprinted Object/(Time by Imprinted Object + Time by Unfamiliar Object).

**Figure 3:**
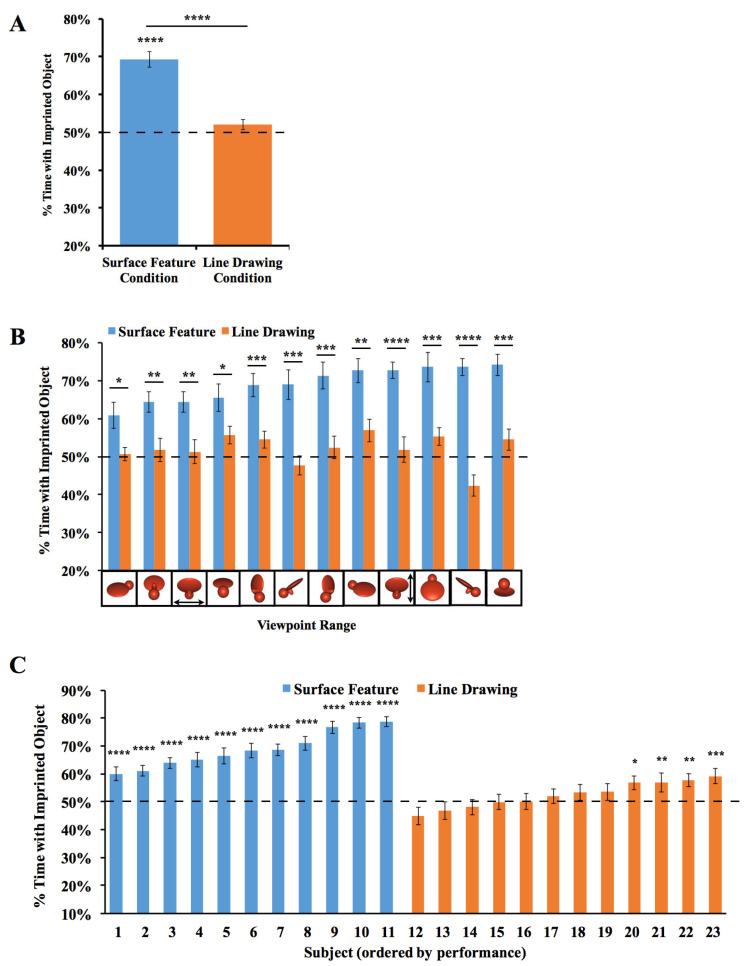
Results from Experiment 1. (**A**) Overall object recognition performance across the test phase. (**B**) Recognition performance on each of the 12 viewpoint ranges. (**C**) Recognition performance of each individual subject. The graphs show the percentage of time spent with the imprinted object versus unfamiliar object. The dashed lines indicate chance performance. Error bars denote ±1 standard error. Asterisks denote statistical significance: * *p* < 0.05; ** *p* < 0.01; *** *p* < 0.001; **** *p* < 0.0001 (two-tailed *t*-tests).

The results are depicted in Figure 3. For each viewpoint range, we computed the percentage of time the chick spent with the imprinted object versus the unfamiliar object. Recognition performance exceeded chance level in the Surface Feature Condition (*t*(10) = 9.75, *p* < 10^−5^, Cohen’s *d* = 2.94), but did not exceed chance level in the Line Drawing Condition (*t*(11) = 1.53, *p* = 0.15, Cohen’s *d* = 0.44).

To test whether performance differed between the Surface Feature Condition and the Line Drawing Condition on each of the test viewpoint ranges, we first used SPSS to compute a repeated-measures ANOVA. We used an ANOVA because we ultimately wanted to run *t*-tests on every viewpoint range, which requires running an overall ANOVA first to control the Type I error rate. We used repeated measures because the viewpoint range varied within subjects. The repeated-measures ANOVA with the viewpoint range as a within-subjects factor and condition (surface feature vs. line drawing) as a between-subjects factor revealed a significant main effect of the viewpoint range (*F*(6.94, 145.81) = 2.73, *p* = 0.01, η_p_^2^ = 0.12) and condition (*F*(1,21) = 52.13, *p* < 0.001, η_p_^2^ = 0.71). The interaction was also significant (*F*(6.94, 145.81) = 2.70, *p* = 0.01, η_p_^2^ = 0.11). Recognition performance was significantly higher in the Surface Feature Condition than the Line Drawing Condition, both in terms of overall recognition performance (*t*(21) = 7.22, *p* < 10^−6^, Cohen’s *d* = 2.99; Figure 3A) and for each of the 12 viewpoint ranges (all *p*s < 0.05, Figure 3B). 

We also examined performance for each of the two imprinted objects. When the chicks were imprinted to Object 1 (see Figure 1 for reference), performance exceeded chance level in the Surface Feature Condition (*t*(4) = 6.54, *p* = 0.003, Cohen’s *d* = 2.92), but not in the Line Drawing Condition (*t*(5) = 1.35, *p* = 0.24, Cohen’s *d* = −0.55). Performance was also significantly higher in the Surface Feature Condition than the Line Drawing Condition for Object 1 (*t*(9) = 7.11, *p* = 0.00006, Cohen’s *d* = 4.13). When the chicks were imprinted to Object 2, performance exceeded chance level in both the Surface Feature Condition (*t*(5) = 7.75, *p* = 0.001, Cohen’s *d* = 3.16) and the Line Drawing Condition (*t*(5) = 5.23, *p* = 0.003, Cohen’s *d* = 2.13), although performance was significantly higher in the Surface Feature Condition than the Line Drawing Condition (*t*(10) = 4.55, *p* = 0.001, Cohen’s *d* = 2.63). In general, newborn chicks developed superior object recognition abilities when reared with objects containing surface features versus line drawings.

Since over 100 test trials were collected from each chick, we could also measure each chick’s object recognition performance with high precision. As shown in Figure 3C, all chicks in the Surface Feature Condition successfully created view-invariant object representations (all *p*s < 0.0001). Conversely, only four of the twelve chicks in the Line Drawing Condition performed above chance level in the task, and those four subjects performed much worse than the subjects in the Surface Feature Condition. 

### 2.3. Discussion

In Experiment 1, newborn chicks developed enhanced object recognition performance when reared with objects containing surface features versus line drawings. Overall, the chicks reared with the line drawings performed at chance level, despite acquiring over 100 h of visual experience with the line drawings during the input phase. Thus, the development of object recognition in newborn chicks requires visual experience with the surface features of objects. 

To verify this conclusion under different testing conditions, we performed a second experiment with two key changes. First, rather than presenting the object from a 60° viewpoint range, the object moved through a 360° viewpoint range. As a result, the chicks were exposed to six times as many unique views of the object during the input phase. Second, we measured each chick’s object recognition abilities with Identity Trials and Viewpoint Trials (Figure 2B). The Identity Trials tested whether the chicks built object representations that were selective for object identity and tolerant to changes in viewpoint. The Viewpoint Trials tested whether the chicks built object representations that were selective for familiar viewpoints. The Identity Trials tested the chicks’ view-invariant object recognition abilities, whereas the Viewpoint Trials tested whether the chicks could use an image-based matching strategy to recognize their imprinted object. 

## 3. Experiment 2

### 3.1. Materials and Methods

The text describing the methods is partly adapted from [4]. The methods were identical to those used in Experiment 1, except in the following ways. First, 20 different subjects were tested. Ten chicks were tested in the Surface Feature Condition [4] and ten chicks were tested in the Line Drawing Condition. No subjects were excluded from the analyses. Second, the imprinted object completed a 360° rotation every 15 s around a frontoparallel vertical axis (see SI Movie 2 for animations). Third, the chicks were tested with Viewpoint Trials and Identity Trials. In the Viewpoint Trials, one display wall showed familiar viewpoints of the imprinted object (rotation around the familiar axis), whereas the other display wall showed novel viewpoints of the imprinted object (rotation around a novel axis, Figure 2B). If the chicks created object representations that were selective for familiar viewpoints, then they should have preferred the imprinted object rotating around the familiar axis over the novel axis. In the Identity Trials, one display wall showed the imprinted object rotating around a novel axis, whereas the other display wall showed a novel object rotating around the familiar axis (Figure 2B). Thus, to recognize their imprinted object in Identity Trials, the chicks needed to build view-invariant representations that were selective for object identity and tolerant to viewpoint changes.

The chicks received 24 test trials per day (168 test trials in total). Figure 2B shows how the objects were presented on the display walls during the input phase and test phase. In total, 6720 h of video footage (14 days × 24 h/day × 20 subjects) were collected for Experiment 2.

### 3.2. Results and Discussion

**Figure 4:**
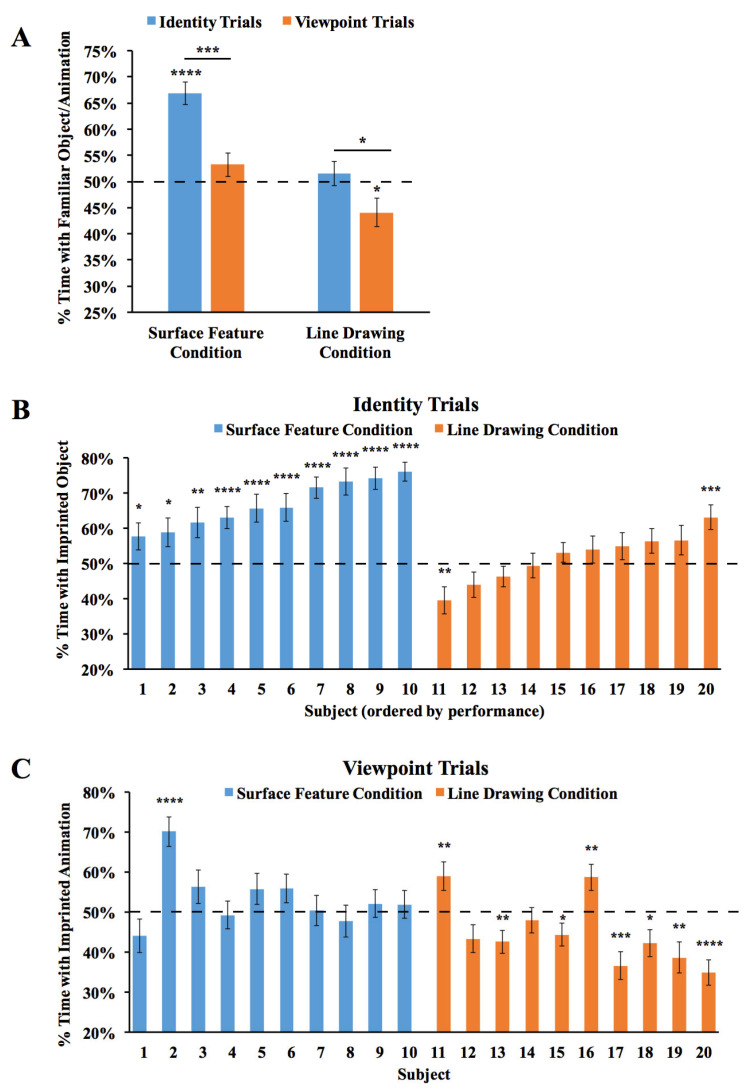
Results from Experiment 2. (**A**) Overall object recognition performance across the test phase. (**B**) Recognition performance of each individual subject in the Identity Trials. (**C**) Recognition performance of each individual subject in the Viewpoint Trials. The dashed lines indicate chance performance. Error bars denote ±1 standard error. Asterisks denote statistical significance: * *p* < 0.05; ** *p* < 0.01; *** *p* < 0.001; **** *p* < 0.0001 (two-tailed *t*-tests).

The results are shown in Figure 4. An ANOVA with the within-subjects factor of the Trial Type (Viewpoint Trials vs. Identity Trials) and the between-subjects factor of the condition (surface feature vs. line drawing) revealed a significant main effect of the condition (*F*(1,18) = 48.55, *p* < 0.001, η_p_^2^ = 0.73), reflecting higher performance in the Surface Feature Condition. The ANOVA also showed a significant main effect of the Trial Type (*F*(1,18) = 14.01, *p* = 0.001, η_p_^2^ = 0.44), reflecting higher performance in the Identity Trials. The interaction was not significant (*F*(1,18) = 1.22, *p* = 0.29, η_p_^2^ = 0.06). In the Surface Feature Condition, performance was above chance level in the Identity Trials (one-sample *t*-test, *t*(9) = 7.84, *p* < 0.001, Cohen’s *d* = 2.48), but not in the Viewpoint Trials (*t*(9) = 1.41, *p* = 0.19, Cohen’s *d* = 0.45). In the Line Drawing Condition, performance did not exceed chance level in the Identity Trials (*t*(9) = 0.70, *p* = 0.50, Cohen’s *d* = 0.22) or the Viewpoint Trials (*t*(9) = 2.22, *p* = 0.053, Cohen’s *d* = 0.70). Thus, when chicks were reared with an object containing surface features, the chicks built object representations that were highly sensitive to identity features. When chicks were reared with line drawings, they did not show evidence for sensitivity to identity or viewpoint features.

We also examined performance for each of the two imprinted objects. We repeated the ANOVA above, but with the addition of the object as a main effect. The ANOVA revealed the same significant effects as before (significant main effects of condition and Trial Type), and the main effect of the object was not significant, nor were the interactions (all *p*s > 0.3). When the chicks were imprinted to Object 1, performance exceeded chance level in the Identity Trials in the Surface Feature Condition (*t*(3) = 4.03, *p* = 0.03, Cohen’s *d* = 2.02), but not in the Line Drawing Condition (*t*(5) = 0.16, *p* = 0.88, Cohen’s *d* = 0.06). In the Identity Trials, performance was significantly higher in the Surface Feature Condition than the Line Drawing Condition (*t*(8) =3.98, *p* = 0.004, Cohen’s *d* = 2.41). Similarly, when the chicks were imprinted to Object 2, performance exceeded chance level in the Identity Trials in the Surface Feature Condition (*t*(5) = 6.44, *p* = 0.001, Cohen’s *d* = 2.63), but not in the Line Drawing Condition (*t*(3) = 0.67, *p* = 0.55, Cohen’s *d* = 0.33). Again, in the Identity Trials, performance was significantly higher in the Surface Feature Condition than the Line Drawing Condition (*t*(8) = 2.68, *p* = 0.03, Cohen’s *d* = 1.64). In the Viewpoint Trials, performance did not exceed chance level when the chicks were imprinted to Object 1 or Object 2 in the Surface Feature Condition or the Line Drawing Condition (*p*s > 0.15).

As shown in Figure 4B, all of the chicks in the Surface Feature Condition exceeded chance level in the Identity Trials (two chicks, *p* < 0.05; one chick, *p* < 0.01; seven chicks, *p* < 0.0001). In contrast, recognition performance was low for all of the chicks in the Line Drawing Condition. Only one chick exceeded chance level in the Identity Trials, while one other chick performed significantly below chance level. As in Experiment 1, newborn chicks developed superior object recognition abilities when reared with objects containing surface features versus line drawings.

### 3.3. Measuring the Strength of the Imprinting Response in Experiments 1 and 2

**Figure 5:**
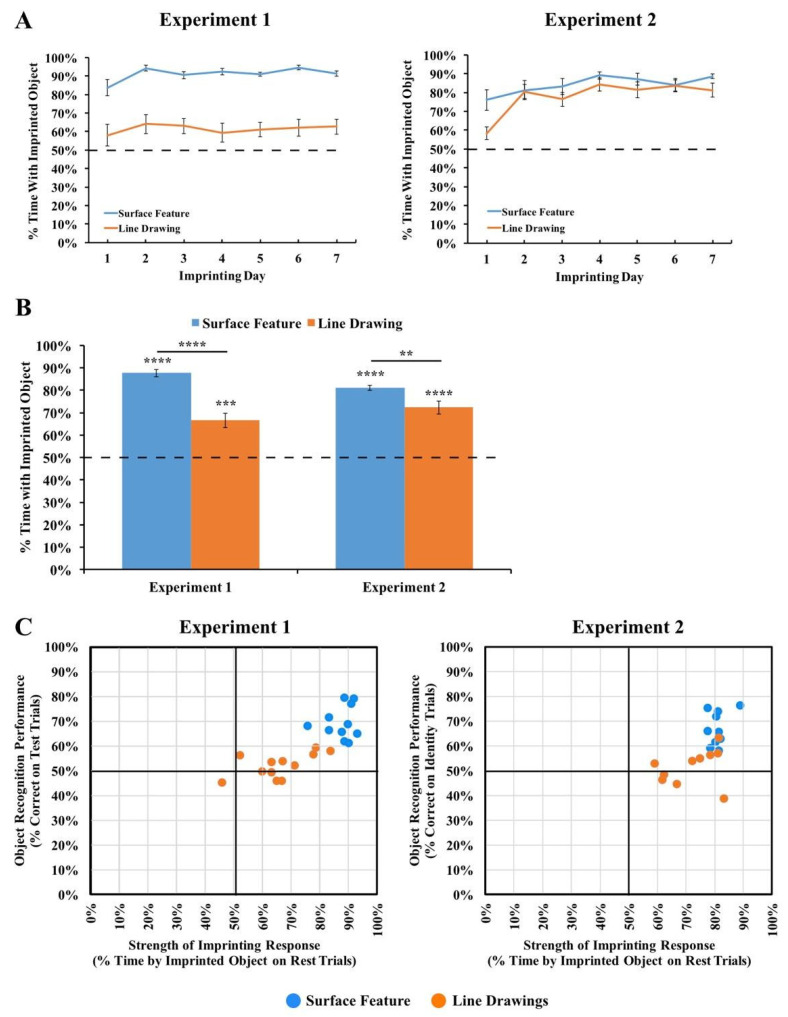
(**A**) Strength of the imprinting response in Experiments 1 and 2 during the input phase. (**B**) Strength of the imprinting response in Experiments 1 and 2 during the rest periods of the test phase. The dashed lines indicate chance performance. Error bars denote ±1 standard error. Asterisks denote statistical significance: ** *p* < 0.01; *** *p* < 0.001; **** *p* < 0.0001 (two-tailed *t*-tests). The chicks successfully imprinted to both the line drawings and the objects with surface features, and this effect was stronger for the objects with surface features. (**C**) Comparison of the chicks’ object recognition performance and the strength of their imprinting response. The chicks developed enhanced object recognition performance when reared with objects with surface features compared to line drawings, even when the chicks imprinted to the objects at similar strengths.

To test the strength of the chicks’ imprinting response, we performed two additional analyses. First, we examined the proportion of time that the chicks spent in proximity to their imprinted object during the input phase. As shown in Figure 5A, the chicks in both Experiments 1 and 2 spent the majority of their time in proximity to the imprinted object during the input phase (Experiment 1 subjects imprinted to surface feature objects: *t*(10) = 40.47, *p* < 10^−11^, *d* = 12.20; Experiment 1 subjects imprinted to line drawings: *t*(11) = 6.27, *p* = 0.00006, *d* = 1.81; Experiment 2 subjects imprinted to surface feature objects: *t*(9) = 20.55, *p* < 10^−8^, *d* = 6.50; Experiment 2 subjects imprinted to line drawings: *t*(9) = 10.84, *p* = 0.000002, *d* = 3.43). Thus, the chicks successfully imprinted to both the line drawings and the objects with surface features. In both experiments, however, the imprinting response was stronger in the Surface Feature Condition than the Line Drawing Condition (Experiment 1: *t*(13.16) = 6.22, *p* = 0.00003, *d* = 2.55; Experiment 2: *t*(18) = 2.15, *p* = 0.05, *d* = 0.96), suggesting that the chicks imprinted less strongly to the line drawings than to the objects with surface features.

Second, we examined the proportion of time the chicks spent with their imprinted object during the rest periods. During the rest periods, the imprinted object was presented on one display wall while the other display wall was blank. The rest periods therefore provided a measure of the strength of the chick’s attachment to the imprinted object during the test phase. As shown in Figure 5B, the chicks in both experiments spent the majority of their time in proximity to the imprinted object during the rest periods (Experiment 1 subjects imprinted to surface feature objects: *t*(10) = 24.91, *p* < 10^−9^, *d* = 7.51; Experiment 1 subjects imprinted to line drawings: *t*(11) = 5.35, *p* = 0.0002, *d* = 1.54; Experiment 2 subjects imprinted to surface feature objects: *t*(9) = 30.14, *p* < 10^−9^, *d* = 9.53; Experiment 2 subjects imprinted to line drawings: *t*(9) = 7.76, *p* = 0.00003, *d* = 2.45). However, the imprinting response was stronger in the Surface Feature Condition than the Line Drawing Condition (Experiment 1: *t*(21) = 5.91, *p* = 0.000007, Cohen’s *d* = 2.50; Experiment 2: *t*(11.30) = 2.89, *p* = 0.01, Cohen’s *d* = 1.29), providing additional evidence that the chicks imprinted less strongly to the line drawings than to the objects with surface features. Importantly, this reduction in the strength of the imprinting response cannot fully explain the low recognition performance because even the chicks that imprinted strongly to the line drawings still built inaccurate object representations (Figure 5C). Together, these analyses suggest that when chicks are reared with line drawings versus objects with surface features, the chicks develop an impaired imprinting response and build less accurate object representations.

## 4. Experiment 3

Experiments 1 and 2 indicate that the development of object recognition requires experience with the surface features of objects. However, there are three limitations to these results. First, the line drawings and the objects with surface features differed in several respects, including their color, contrast, hue homogeneity, and complexity. Thus, it is unclear which particular features caused the observed differences in recognition performance across the conditions. Indeed, there is extensive evidence that color is one of the most distinctive features encoded in imprinting (e.g., [30,44,45,46,47,48]), which raises the possibility that color differences may have influenced performance across the conditions. Second, Experiments 1 and 2 tested chicks’ recognition performance with the same two objects, so it is unclear whether these results generalize to other objects. Third, the imprinting response was less strong in the Line Drawing Condition than the Surface Feature Condition, potentially because of the color differences across objects. Given that recognition performance in this task is directly constrained by the strength of the imprinting response, the weaker imprinting response in the Line Drawing Condition likely produced lower recognition performance in the test trials.

**Figure 6:**
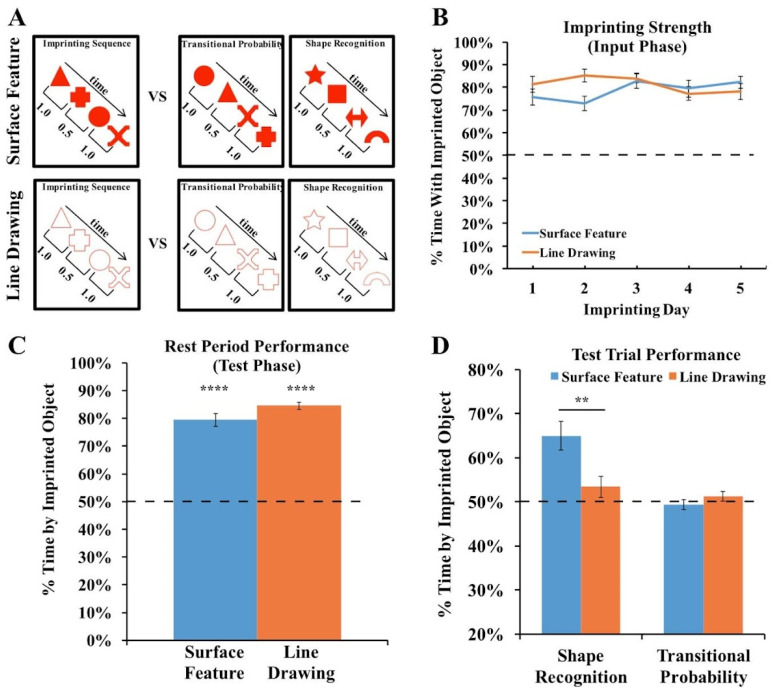
(**A**) Experiment 3 method. During the input phase, an imprinting sequence defined by the transitional probabilities (TPs) within and between shape pairs appeared on one display wall at a time. The imprinting sequence either contained shapes with surface features or line drawings of shapes. During the test phase, we presented chicks with two-alternative forced-choice tasks. In each test trial, one display wall showed the imprinting sequence, and the other display wall showed either the same shapes as in the imprinting sequence but in novel orders (TP trials) or a sequence of novel shapes (shape recognition trials). (**B**) Strength of the imprinting response in Experiment 3 during the input phase. (**C**) Strength of the imprinting response in Experiment 3 during the rest periods of the test phase. The chicks imprinted equally strongly across the Surface Feature and Line Drawing Conditions. (**D**) Recognition performance in the Shape Recognition and Transitional Probability Conditions. The chicks showed superior object recognition performance in the Surface Feature Condition compared to the Line Drawing Condition. The dashed lines indicate chance performance. Error bars denote ±1 standard error. Asterisks denote statistical significance: ** *p* < 0.01; **** *p* < 0.0001 (two-tailed *t*-tests).

To provide a more direct comparison of chicks’ recognition performance across conditions, we performed a third experiment in which the objects in the Surface Feature and Line Drawing Conditions were the same color (red). We also ensured that the objects did not have shadows and regions with different luminance values (as in Experiments 1 and 2), by using two-dimensional objects, rather than three-dimensional objects. The only difference between the conditions was whether the objects did, or did not, have surface features (Figure 6A).

For the Surface Feature Condition, we used the data previously reported in [42]. In that paper, we tested whether newborn chicks can encode the transitional probabilities (TPs) between shapes in a sequence. During the input phase, the chicks were reared with an imprinting sequence consisting of a stream of four shapes, and the order of the shapes was defined by the TPs within and between shape pairs. During the test phase, we presented two types of test trials. In the shape recognition trials, one monitor showed a sequence of familiar shapes, and the opposite monitor showed a sequence of novel shapes. In the TP trials, both monitors showed the familiar shapes, but we manipulated the TPs between shapes. One monitor showed a familiar TP sequence, in which the TPs between shapes matched the imprinting sequence, and the opposite monitor showed a novel TP sequence, in which the TPs between shapes did not match the imprinting sequence. In the original study, we found that the chicks successfully distinguished between the sequences in the shape recognition trials, but failed to distinguish between the sequences in the TP trials. Here, we repeated this experiment with one crucial change: rather than presenting sequences of shapes with surface features, we presented sequences of red line drawing shapes (Figure 6A). 

### 4.1. Materials and Methods

For a detailed description of the methods, see [42]. In the present study, we used a similar design to the original study, except that the chicks were imprinted and tested with red line drawings, rather than red objects with surface features. As in the original study, we tested the chicks with both shape recognition and TP test trials. We tested 12 subjects in the Surface Feature Condition [42] and 10 subjects in the Line Drawing Condition. No subjects were excluded from the analyses.

### 4.2. Results and Discussion

We first examined the strength of the imprinting response to ensure that the chicks imprinted equally strongly across the two conditions. As shown in Figure 6B, the chicks in both conditions spent the majority of their time in proximity to the imprinted object during the input phase (one-sample *t*-tests, Surface Feature Condition: *t*(11) = 12.35, *p* < 10^−7^, Cohen’s *d* = 3.57; Line Drawing Condition: *t*(11) = 18.77, *p* < 10^−8^, Cohen’s *d* = 5.42). Unlike in Experiments 1 and 2, the chicks did not show a stronger imprinting response in the Surface Feature Condition than the Line Drawing Condition (independent samples *t*-test, *t*(22) = 0.7, *p* = 0.94, Cohen’s *d* = 0.03). Similarly, as shown in Figure 6C, the chicks in both conditions spent the majority of their time in proximity to the imprinted object during the rest periods (one-sample *t*-tests, Surface Feature Condition: *t*(11) = 13.06, *p* < 10^−7^, Cohen’s *d* = 3.77; Line Drawing Condition: *t*(9) = 28.59, *p* < 10^−10^, Cohen’s *d* = 9.04). Unlike in Experiments 1 and 2, the chicks did not show a stronger imprinting response during the rest periods in the Surface Feature Condition than the Line Drawing Condition (independent samples *t*-test, *t*(20) =1.69, *p* = 0.11, Cohen’s *d* = 0.75). Together, these analyses show that the chicks did not imprint more strongly in the Surface Feature Condition, allowing for a more direct comparison of recognition performance across the conditions.

The chicks’ recognition performance is shown in Figure 6D. An ANOVA with the within-subjects factor of the Trial Type (shape recognition vs. TP trials) and the between-subjects factor of the condition (surface feature vs. line drawing) revealed a significant main effect of the Trial Type (*F*(1,20) = 15.95, *p* = 0.001, η_p_^2^ = 0.44), a significant main effect of the condition (*F*(1,20) = 4.38, *p* = 0.049, η_p_^2^ = 0.18), and a significant interaction between the Trial Type and condition (*F*(1,20) = 9.16, *p* = 0.007, η_p_^2^ = 0.31). 

When reared with a sequence of shapes containing surface features, the chicks could reliably distinguish between familiar shapes and novel shapes (one-sample *t*-test, *t*(11) = 4.67, *p* = 0.0007, Cohen’s *d* = 1.35). In contrast, when reared with a sequence of line drawing shapes, the chicks failed to distinguish between familiar shapes and novel shapes (one-sample *t*-test, *t*(9) = 1.30, *p* = 0.23, Cohen’s *d* = 0.41). As in the original study [42], the chicks in both conditions failed to distinguish between the sequences based on the TPs between shapes (Surface Feature Condition: *t*(11) = 0.56, *p* = 0.59, Cohen’s *d* = −0.16; Line Drawing Condition: *t*(9) = 1.04, *p* = 0.33, Cohen’s *d* = 0.33). 

On the individual-subject level, 8 of the 12 chicks in the Surface Feature Condition showed a statistically significant preference for the familiar shapes (7 chicks: *p* < 0.0001; 1 chick: *p* < 0.05). In contrast, in the Line Drawing Condition, only one chick exceeded chance level in the shape recognition trials, while one other chick performed significantly below chance level. As in Experiments 1 and 2, newborn chicks developed superior object recognition performance when reared with objects containing surface features versus line drawings.

## 5. General Discussion

A deep understanding of object recognition requires understanding the role of visual experience in development. Here, we reveal a set of conditions under which object recognition fails to develop in newborn animals: when a newborn’s visual experience with objects consists solely of line drawings. When newborn chicks were reared with objects containing surface features, the chicks developed robust view-invariant object recognition. In contrast, when chicks were reared with line drawings of objects, the chicks failed to develop object recognition. Notably, the chicks reared with the line drawings performed at chance level, despite acquiring over 100 h of experience with the objects. Thus, the development of object recognition requires visual experience with the surface features of objects.

Interestingly, the chicks reared with line drawings failed to build accurate object representations despite being raised in environments that contained some surface features. The walls and floor of the chamber contained surface features, as did the heaps of grain consumed during feeding. Nevertheless, when the *objects* in the chicks’ visual environment lacked surface features, the chicks failed to build accurate representations. This finding suggests that experience with surface features per se is not sufficient for the development of object recognition; rather, newborn visual systems need experience with the surface features of objects. 

These results add to a growing body of work mapping out the conditions under which object recognition does, and does not, emerge in newborn animals. For instance, studies with newborn chicks have revealed two constraints on the development of object recognition. First, there is a “slowness constraint” on newborn vision: object recognition emerges when newborn chicks are reared with slowly moving objects, but not quickly moving objects [4]. When chicks are reared with quickly moving objects, their object representations become distorted in the direction of object motion and fail to generalize to novel viewpoints and rotation speeds. Second, there is a “smoothness constraint” on newborn vision: object recognition emerges when newborn chicks are reared with temporally smooth objects, but not temporally non-smooth objects [3,49]. When chicks are reared with temporally non-smooth objects, their object representations are less selective for object identity. The present study extends this literature by demonstrating that experience with slow and smooth objects is not sufficient for the development of object recognition. The line drawings in Experiments 1–3 moved slowly and smoothly over time, but the chicks nevertheless failed to develop object recognition. Together, these findings indicate that the development of object recognition requires experience with naturalistic objects: objects that have surface features and move slowly and smoothly over time. 

More generally, these results build on a large body of research using animal models to examine the mechanisms of object recognition and early visual learning. For decades, newborn chicks have been used to characterize the effects of visual experience on the brain (e.g., [50,51,52]) and to isolate the neural mechanisms that underlie imprinting (e.g., [53,54]). Studies of chicks have also revealed predispositions that might shape early visual learning (e.g., [55,56,57]). Another important animal model for studying early visual learning is rodents. Studies of rats provide converging evidence that high-level vision is not unique to primates. Like newborn chicks, rats can recognize objects across novel viewpoints (e.g., [43]). Normal visual development in rats also requires experience with a slow and smooth visual world [58], suggesting that avian and mammalian brains are subject to common developmental constraints. Specifically, when newborn rats were reared with frame-scrambled versions of natural movies (which preserved the natural spatial statistics but resulted in quickly changing, temporally unstructured input), the rats developed fewer complex cells in the primary visual cortex, the cells showed abnormally fast response dynamics, and the cells were less likely to support stable decoding of stimulus orientation. Thus, depriving newborn animals of slowly changing visual experiences disrupts normal visual development in both birds and mammals, potentially reflecting a shared cortex-like canonical circuit found across taxa [37]. 

### Limitations of This Study and Directions for Future Research

While these results contribute to our understanding of early visual development, there are limitations to this work that will require additional future research. First, these chicks were reared with either one 3D object (Experiments 1 and 2) or four 2D objects (Experiment 3). It is therefore possible that chicks could develop object recognition in a visual world consisting solely of line drawings if there were more line drawings in the environment and/or if those line drawings were more complex (e.g., line drawings containing polyhedral shapes that included L, Y, and T junctures between lines). Future studies could distinguish between these possibilities by rearing chicks in more complex “line drawing worlds”.

Second, we used the chicks’ preference for their imprinted object as a measure of object recognition performance. While successful performance provides evidence for object recognition (e.g., in the Surface Feature Conditions), the absence of a preference (e.g., in the Line Drawing Conditions) does not necessarily provide evidence for a lack of object recognition. For instance, it is possible that the chicks in the Line Drawing Conditions perceived the test objects as different but grouped them in the same object category. Of course, this alternative explanation must then explain why the presence of surface features would lead chicks to categorize objects differently from one another, whereas line drawings would lead chicks to categorize objects together. It would be interesting for future studies to test chicks using alternative methods (e.g., reinforcement learning) to explore whether the present findings generalize to other object recognition tasks. 

Third, these results do not reveal why surface features are necessary for the development of object recognition. Why do newborn chicks fail to understand line drawings, when mature animals (including birds) can readily recognize objects presented in line drawings? One possibility is that the mechanisms underlying object recognition require patterned input from natural visual objects in order to develop a receptive field structure that efficiently recovers edges and lines. Specifically, in natural visual environments, the edges of objects and surfaces are typically marked by discrete changes in surface attributes, and mature visual systems contain neurons tuned to the orientation of these contours, responding to edges and lines [59,60]. To develop these orientation-tuned detectors, newborn brains may require visual experience of objects with surface features. 

Moreover, line drawings are impoverished compared to real objects, and the surface features that appear on real objects may provide valuable information for building accurate representations of an object’s three-dimensional shape. For example, the surface features on the objects used in Experiments 1 and 2 had gradients of luminance that moved as the object rotated, creating flow field cues for three-dimensional shape. Accordingly, it is a possibility that experience with realistic objects is necessary to develop the ability to recognize objects in line drawings. Future controlled-rearing experiments could test this hypothesis directly by examining whether experience with realistic objects allows for the development of line drawing understanding.

Ultimately, a deep mechanistic understanding of the role of experience in the development of object recognition will require task-performing computational models that can simulate the complex interactions between newborn brains and the visual environment. The present results should be valuable for this enterprise because they provide precise descriptions of how specific visual inputs relate to specific object recognition outputs in a newborn model system. These input–output patterns can serve as benchmarks for measuring the accuracy of computational models (e.g., [61,62,63,64]). Specifically, to explain the development of object recognition, a computational model would need to produce two patterns. First, the model should successfully develop view-invariant object recognition when trained with realistic objects that move slowly and smoothly over time. Second, the model should fail to develop object recognition when trained solely with line drawings.

## 6. Conclusions

The present study provides evidence that the development of object recognition requires experience with the surface features of objects. Newborn chicks develop enhanced object recognition performance when reared with objects containing surface features compared to line drawings. This study sheds light on how a fundamental ability emerges in newborn animals and provides precise input–output patterns for measuring the accuracy of task-performing computational models of visual development. Furthermore, these insights into the role of experience in object recognition may offer valuable parallels to understanding and potentially enhancing early visual development in human infants.

## Data Availability

Data are contained within the article and Supplementary Materials.

## Supplementary Materials

The following supporting information can be downloaded at: https://www.mdpi.com/article/10.3390/ani14020284/s1, Movie S1: Experiment 1 Imprinting Stimuli; Movie S2: Experiment 2 Imprinting Stimuli. Data S1: Chick Performance Data for Experiments 1–3.
